# A Rare Case of Vulvar Hibernoma Treated With Resection

**DOI:** 10.7759/cureus.9111

**Published:** 2020-07-10

**Authors:** Jason M Zhao, Dawood Tafti, Erica Kao, Ryan Schwope

**Affiliations:** 1 Department of Radiology, San Antonio Military Medical Center, San Antonio, USA; 2 Department of Pathology, San Antonio Military Medical Center, San Antonio, USA

**Keywords:** hibernoma, liposarcoma, lipoma

## Abstract

Hibernoma is a rare benign neoplasm of brown adipose tissue most frequently involving the thigh, shoulder, back, and neck. Differentiating this benign entity from other lipomatous tumors such as well-differentiated liposarcoma is essential, given the different surgical approaches and prognosis associated with each diagnosis. It is helpful for the radiologist to recognize the uncommon locations of hibernoma, as well as characteristic imaging features, in order to properly include it in the differential considerations. Here we present a rare case of symptomatic vulvar hibernoma in a 25-year-old woman treated with surgical excision.

## Introduction

Hibernoma is a rare benign neoplasm of brown adipose tissue accounting for <2% of benign lipomatous tumors and 1% of all adipocytic tumors [1]. It has a peak incidence in the third decade of life with a slight male predominance [2]. The majority of patients present with a painless, slowly growing, soft tissue mass which is mobile, pliable, and may feel warm due to its hypervascularity [3]. In a comprehensive morphologic analyses of 170 hibernomas from the soft tissue registry of the Armed Forces Institute of Pathology (AFIP), the authors found that hibernomas most commonly occur in the thigh (29%), followed by the shoulder (12%), back (10%), neck (9%), and chest wall (6%) [2]. Other uncommon anatomic locations were also reported sporadically in case reports and small case series to include intraosseous spaces, breasts, mediastinum, and adrenal glands [4-8]. Vulvar hibernoma is exceedingly rare with only one reported case in the literature [9]. Here we present a case of symptomatic vulvar hibernoma in a 25-year-old woman treated with surgical resection.

## Case presentation

**Figure 1:**
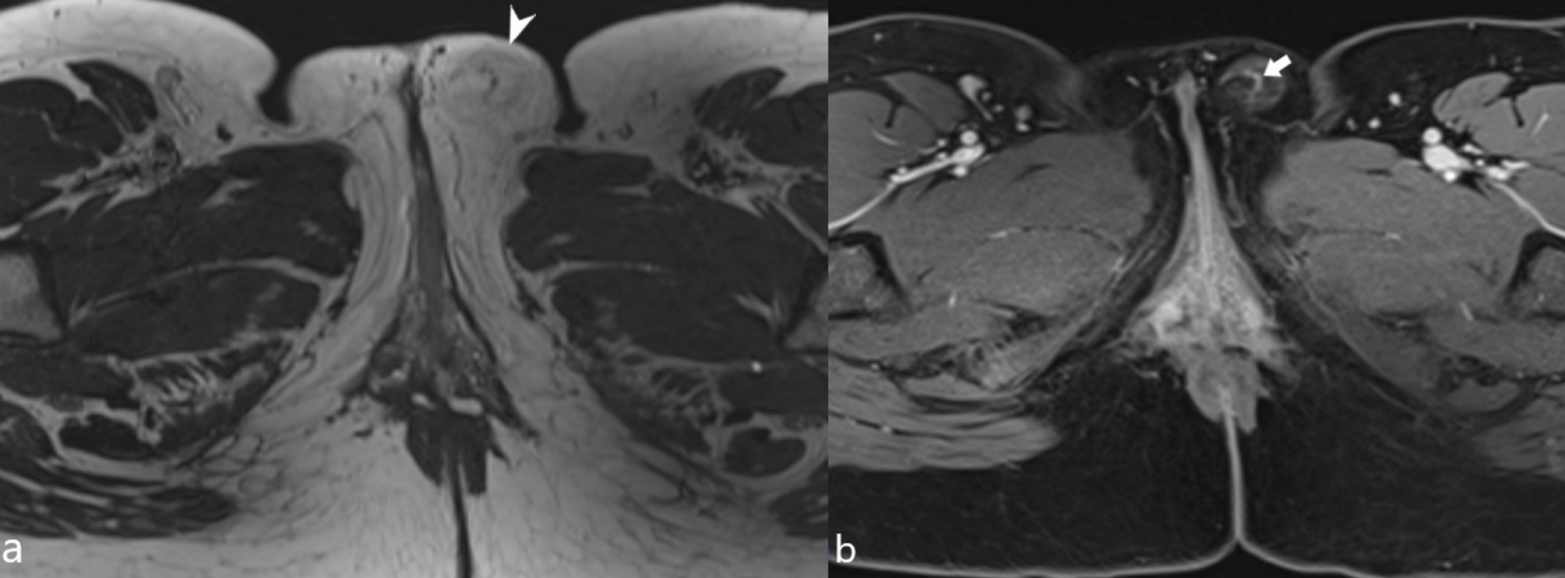
MRI images of a vulvar hibernoma (a) Pre-contrast T1-weighted MRI shows a circumscribed, lipomatous mass (white arrowhead) within the left vulva with mild T1 hypointensity compared to subcutaneous fat. (b) Post-contrast, fat saturation, T1-weighted MRI shows heterogeneous enhancement with a prominent vessel (white arrow) coursing through the central portion of the tumor.

A 25-year-old G2P1 woman with a history of hernia repair presented with a slowly enlarging left labial mass, first noticed approximately eight years ago when she felt intermittent sharp and burning pain in the region aggravated by wearing tight pants. The mass was visible on the physical exam and measured up to 5 cm in size. Sporadic exacerbation of pain was frequently noted without exacerbating factors; however, it was also occasionally noted when wearing tight pants. Follow-up pelvic MRI showed a circumscribed, lipomatous mass in the subcutaneous soft tissues of the left labia measuring 4.9 cm x 2.1 cm x 1.6 cm (Figure 1). The mass appears mildly T1 hypointense to the adjacent subcutaneous fat and hyperintense to the surrounding muscles; there is Indian-ink chemical shift artifacts on the opposed-phase gradient recalled echo (GRE) images, confirming macroscopic fat within the lesion; there is also heterogeneous T1 enhancement on the post-contrast, fat-saturated T1 sequence; a prominent vessel courses through the central portion of the mass.

**Figure 2:**
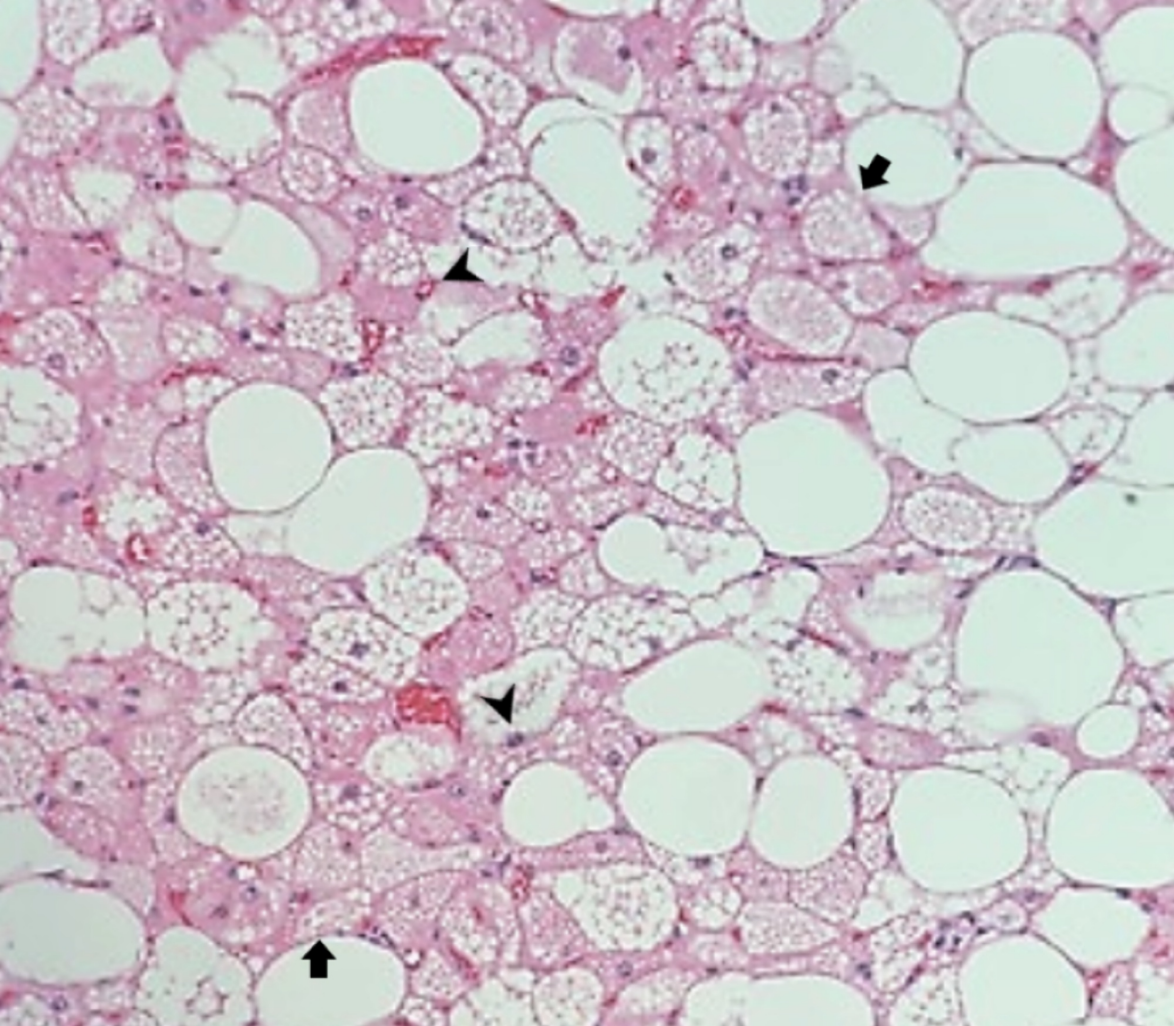
Histologic image of a vulvar hibernoma Hematoxylin and eosin stain analysis of the vulvar lipomatous mass demonstrates clusters of microvacuolated brown fat cells (black arrows) with eosinophilic granular cytoplasm (black arrowheads) representative of hibernoma cells (×200 magnification).

The vulvar mass was surgically excised and sent for histopathologic analysis. The gross surgical specimen was well circumscribed with a tan and homogenous cut surface. On histological examination, the tissue showed clusters of microvacuolated brown fat cells with eosinophilic granular cytoplasm (hibernoma cells) without malignant features, which are characteristic of typical hibernoma (Figure 2). 

## Discussion

Hibernoma is a rare benign neoplasm composed of brown lipomatous tissues without the potential for malignant transformation. The term “hibernoma” was coined in 1914 by Gery and came from the relatively common occurrence of brown fat in hibernating animals [3]. The majority of hibernomas exhibit slow growth without inciting symptoms, often presenting as a painless swelling in the subcutaneous soft tissues and incidentally discovered on imaging. Thus, the sharp pain associated with our patient’s vulvar hibernoma is an atypical clinical presentation, which may be attributed to the network of rich superficial nerve endings in the vulvar region. The previously noted case of a vulvar hibernoma was described in a 35-year-old female presenting with a painless subcutaneous mass of the mons pubis and was also treated uneventfully with surgical excision [9].

Hibernomas are composed of various proportions of brown fat cells with eosinophilic granular cytoplasm and microvacuolation (hibernoma cells), multivacuolated adipocytes, and mature univacuolated adipocytes. Four morphologic variants of hibernomas have been described: typical (82%), myxoid (8%), lipoma-like (7%), and spindle cell (2%) [2]. Typical hibernomas are by far the most common subtype and contain mainly classic, multivacuolated brown fat cells: the current case is a great example (Figure 2). The myxoid subtype is notable for myxoid degeneration of the stroma and affects primarily males. The lipoma-like subtype predominantly contains mature, white fat cells with scattered granular multivacuolated adipocytes (brown fat cells). Finally, the spindle cell subtype most commonly occurs in the neck and has combined features of hibernoma and spindle cell lipoma to include bland spindle cells, ropey collagen, mast cells, and myxoid stroma. 

It is important to be aware of these rarer subtypes of hibernoma as the lipoma-like subtype of hibernoma could be mistaken for atypical lipomatous tumor/well-differentiated liposarcoma (ALT/WDLS) due to the presence of scattered multivacuolated adipocytes mimicking lipoblasts [2]. The intra-abdominal location of these tumors and heterogeneous appearance on the contrast-enhanced CT or MRI are also features associated with WDLS, potentially adding to the diagnostic complexity. Recently, Al Hmada et al. analyzed 64 ALT-like hibernoma cases and found histological features that favor hibernoma over ALT, including small nuclei without hyperchromasia or atypia, smaller vacuoles than true lipoblasts with minimal scalloping of nuclei, more numerous lipoblast-like cells as opposed to scattered true-lipoblasts in ALT/WDLS, and presence of adipocytes with granular eosinophilic cytoplasm [10].

Hibernomas classically appear as a circumscribed, lobulated, lipomatous mass on MRI, demonstrating mild T1 hypointensity to subcutaneous fat, incomplete fat suppression on short TI inversion-recovery (STIR) sequences, and heterogeneous enhancement. There may be enhancing internal septations and vascularity within the lesion: in fact, branching flow voids in non-contrasted studies and enhancing vascular structures are considered a distinguishing feature that favors hibernoma over WDLS [3]. Differentiating hibernoma from simple lipoma on MRI is straightforward because the latter typically shows homogeneous T1 hyperintensity, signal loss on fat suppression, and uniform contrast enhancement pattern.

Differentiating hibernoma from ALT/WDLS by imaging before tissue diagnosis can be a diagnostic dilemma. Both entities can be well circumscribed and show T1 hypointensity to the surrounding fat and incomplete fat saturation; both may contain thickened (>3 mm) and enhancing septation. Individual hibernoma of certain subtype may exhibit unique imaging characteristics. For example, the lipoma-like and spindle-cell subtypes may show internal serpiginous vascular structures, while the myxoid subtype may have non-specific high T2 signals due to its high water content [3]. In contrast, WDLS often have an irregular capsule with decreased vascularity, sometimes with areas of mineralization which is not seen in hibernomas. In addition, more aggressive ALT can also show malignant behaviors, such as locoregional invasion, lymphadenopathy, and metastasis. When imaging fails to distinguish a benign hibernoma from a potentially malignant ALT, surgical excision remains the definitive diagnosis and treatment of choice.

Other modalities including positron emission tomography (PET) have also been utilized to characterize hibernomas. Several studies have attempted to use standardized uptake values (SUV) in 18-fluoro-2-deoxy-D-glucose (18FDG)-PET to differentiate between hibernoma and other ALTs. For example, hibernoma was shown to have intense FDG uptake greater than 7.9, while liposarcomas have low to intermediate uptake values between 0.8 and 6.0 depending on the tumor grade; simple lipomas usually have low uptake values less than 2.0 [11-15]. However, subsequent case reports showed intermediate SUV less than 6.7 for hibernomas, overlapping with the range of liposarcoma uptake values [16,17]. These findings suggest different subtypes of hibernoma may show different FDG metabolic activities depending on the proportions of their brown fat contents. Smith et al. in their analyses of three incidental hibernomas further postulated that the fluctuations in SUVs, rather than the actual SUVs, maybe a better biomarker for hibernoma [17].

The definitive and curative treatment of hibernoma is surgical excision. No recurrence of hibernoma after excision has been reported in the literature. For patients who cannot tolerate surgery, routine surveillance may be considered. When an ALT is encountered in daily practice, the imaging features outlined above can be used to distinguish hibernomas from more suspicious entities such as WDLS. FDG-PET may offer additional information to characterize the brown fat content of the tumor, but the SUVs are often non-specific for the diagnosis of hibernoma and histopathologic diagnosis remains the gold standard.

## Conclusions

Hibernomas are rare benign neoplasms of brown adipose tissue. The primary differential diagnosis of a hibernoma on imaging is a liposarcoma due to their shared hypervascularity. We report a hibernoma of the vulva which is an exceedingly rare location for this lesion with only one other reported case in the literature. Although MRI plays an important role in establishing a differential diagnosis, surgical resection remains the gold standard in order to definitively exclude a liposarcoma.
